# Rehabilitation through virtual reality: physical activity of patients admitted to the intensive care unit

**DOI:** 10.5935/0103-507X.20190078

**Published:** 2019

**Authors:** Tamires Teixeira Gomes, Debora Stripari Schujmann, Carolina Fu

**Affiliations:** 1 Departamento de Fisioterapia, Fonoaudiologia e Terapia Ocupacional, Faculdade de Medicina, Universidade de São Paulo - São Paulo (SP), Brasil.

**Keywords:** Physical activity, Video games, Virtual reality, Physical therapy modalities, Intensive care units

## Abstract

**Objective:**

To evaluate the level of activity that Nintendo Wii^TM^ can elicit in intensive care unit patients and its associated safety and patient satisfaction.

**Methods:**

Experimental, single-center study performed at a tertiary care hospital. Patients ≥ 18 years old who were admitted to the intensive care unit, participated in videogames as part of their physical therapy sessions and did not have mobility restrictions were included. Th exclusion criteria were the inability to comprehend instructions and the inability to follow simple commands. We included n = 60 patients and performed 100 sessions. We used the Nintendo Wii^TM^ gaming system in the sessions. An accelerometer measured the level of physical activity of patients while they played videogames. We evaluated the level of activity, the modified Borg scale scores, the adverse events and the responses to a questionnaire on satisfaction with the activity.

**Results:**

One hundred physical therapy sessions were analyzed. When the patients played the videogame, they reached a light level of activity for 59% of the session duration and a moderate level of activity for 38% of the session duration. No adverse events occurred. A total of 86% of the patients reported that they would like to play the videogame in their future physical therapy sessions.

**Conclusion:**

Virtual rehabilitation elicited light to moderate levels of activity in intensive care unit patients. This therapy is a safe tool and is likely to be chosen by the patient during physical therapy.

## INTRODUCTION

Inactivity is characterized by low mobility and the absence of physical activity.^(1)^ Studies from several countries have shown that few patients in the intensive care unit (ICU) are physically active. A very small percentage of patients have reached a high level of intensity during physical activity. These data showed that the level of activity of patients when they are in the ICU is very low.^(2-4)^ Inactivity can cause respiratory and cardiac issues and musculoskeletal injuries.^(5-7)^ In addition, these patients may exhibit changes in balance and coordination as well as *delirium* due to sensory deprivation of visual, auditory, and tactile stimuli.^(8)^ These alterations might impact patient functionality, as previous studies have shown a posthospitalization reduction in functionality, leading to a reduced quality of life.^(9,10)^

Given the alterations that inactivity can cause in ICU patients, early and progressive rehabilitation programs with high levels of activity have been developed for ICU patients.^(11-13)^ Recent studies have investigated alternative methods that can be used during physical therapy to complement traditional treatment and offer a sufficient level of activity to reverse the state of inactivity.^(14-16)^ In other environments, interactive games that use virtual reality in physical therapy sessions have previously been proposed as therapeutic options.^(4,17-19)^ Virtual reality technology simulates games and sports and use a controller to capture movement that is shown on a screen.^(17,20)^ Studies have shown that game systems can generate sufficient movement to produce physical activity and help to improve the balance, mobility, muscle strength, and cognition of older adults. This technology is beneficial not only because it addresses the components of rehabilitation that are directly involved in physiopathology but also because it stimulates patients' interest and motivation regarding therapy.^(21-25)^

Thus, the use of virtual reality can also be a tool for rehabilitation and exercise for patients in intensive care.^(20,26)^ Few studies on ICU patients in the literature have investigated the use of this tool, its safety and feasibility, and the level of activity it can offer patients. Therefore, we hypothesize that virtual reality can be used as a new tool in the rehabilitation of this patient population because it is safe, practical, and capable of eliciting a level of activity in critically ill people. To test this hypothesis, this study aimed to evaluate the safety and feasibility of virtual reality as a rehabilitation tool for patients in the ICU as well as assess the level of physical activity that it is able to elicit in these patients when they are using it.

## METHODS

This was an experimental study. The study took place in the ICU of the Emergency Department of the *Instituto Central* of *Hospital das Clínicas, Faculdade de Medicina, Universidade de São Paulo*. The institution's Ethics Committee approved this study (approval number 662.187).

During the period between November 2016 and January 2018, a randomized clinical trial, "Progressive Mobility Program and Technology to Improve the Level of Physical Activity and Functionality of ICU Patients" (NCT 02889146), was conducted in the ICU. The rehabilitation program included a video game session.

This study is a secondary analysis of the main trial. We analyzed and described the patients who were in the intervention group and participated in the exercise sessions with video games. Thus, patients who participated in the program group and performed this intervention were analyzed regarding the objectives of this specific study. For the video game sessions during the program, patients who were aged ≥ 18, did not have a neurological pathology, did not present a skin condition that could prevent the use of the accelerometer, and did not have any mobility restrictions were considered eligible for the study. The exclusion criteria were the inability to comprehend instructions for the videogames and the inability to follow simple commands.

### Assessment

#### Demographic and clinical information

For sample characterization, the sex, age, Simplified Acute Physiology Score III (SAPS III), Charlson comorbidity index, and Glasgow coma score of the patients and the surgical procedures were collected from the patients' charts.

#### Safety

The vital signs were assessed before the intervention began, immediately after the session ended, and after a five-minute rest period. The vital signs were reviewed to detect any significant differences in the signs from the safety criteria for mobilization in critically ill patients or activity interruption criteria outlined in the literature, as well as any intercurrence.^(27,28)^ The presence of a catheter, tube, drain, probe, or oxygen therapy device, as well as the use of a vasoactive drug or invasive mechanical ventilation (IMV) at the moment of therapy, was also recorded. Additionally, adverse events, including the accidental removal of these lines and the need to increase medication or oxygen supply, were recorded.

### Assessment of the level of activity

#### Accelerometry

The level of physical activity was evaluated using an activity monitor with a tri-axial accelerometer; it was placed on the wrist and ankle of the patient while they played a video game. The ActiGraph GT3X monitor has been validated and is used to measure activity objectively.^(29,30)^ This monitor can detect changes in acceleration in three axes, the vertical, horizontal, and perpendicular axes, creating a continuous recording of minimal movements.

The monitor provides specific information, such as the percentage of time spent in different levels of activity: light activity, moderate activity, vigorous activity, and very vigorous activity. Thus, it collected the maximum level of physical activity achieved by the patient during the videogame session and the duration of time the patient spent in each activity level. The data were analyzed with software provided by the manufacturer of the monitor (ActiLife 6). Using a validated algorithm,^(31)^ the duration of each of the activity levels was determined for each patient.

#### Modified Borg scale

The modified Borg scale is a scale from 0 to 10 that assesses respiratory fatigue, where 0 corresponds to no fatigue and 10 corresponds to the most fatigue the subject has ever felt.^(32,33)^ It is a subjective scale, so it is necessary to evaluate the patient's understanding when classifying respiratory fatigue.^(32,33)^ This scale presented good reproducibility when evaluating respiratory fatigue during the exercise.^(32)^ Therefore, before starting the game, we explained this instrument to the patient and then asked the patient to classify his or her level of respiratory fatigue.

#### Satisfaction

A questionnaire assessing patients' satisfaction with the videogame session was administered to the patient at the end of the session. To evaluate the patients' perception regarding the videogame sessions, we used a simple questionnaire with five questions developed by the study researchers and based on a satisfaction questionnaire found in the literature.^(34)^ The following questions were asked:

1. Do you think you are able to play this game?
2. On a 0 to 10 scale, how much did you like this activity?
3. Do you prefer the videogame session or other activities during physical therapy? Which activity do you prefer to do?
4. Would you like to take on this activity in the next physical therapy session?
5. Did you feel any discomfort during this activity? What was it?

#### Intervention

**Figure 1:**
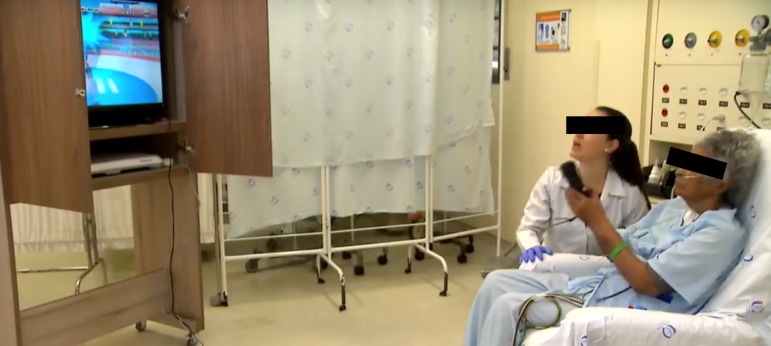
Patient receiving instructions on how to play the game shown on the television during the physiotherapy session. Patients sitting on a chair playing the game were properly monitored.

The videogame sessions were held during physical therapy sessions by a physiotherapist who was trained to administer the games. Patients received instructions about how to play the game, and before starting the game, they watched the physiotherapist play one game (Figure 1).

**Figure 2:**
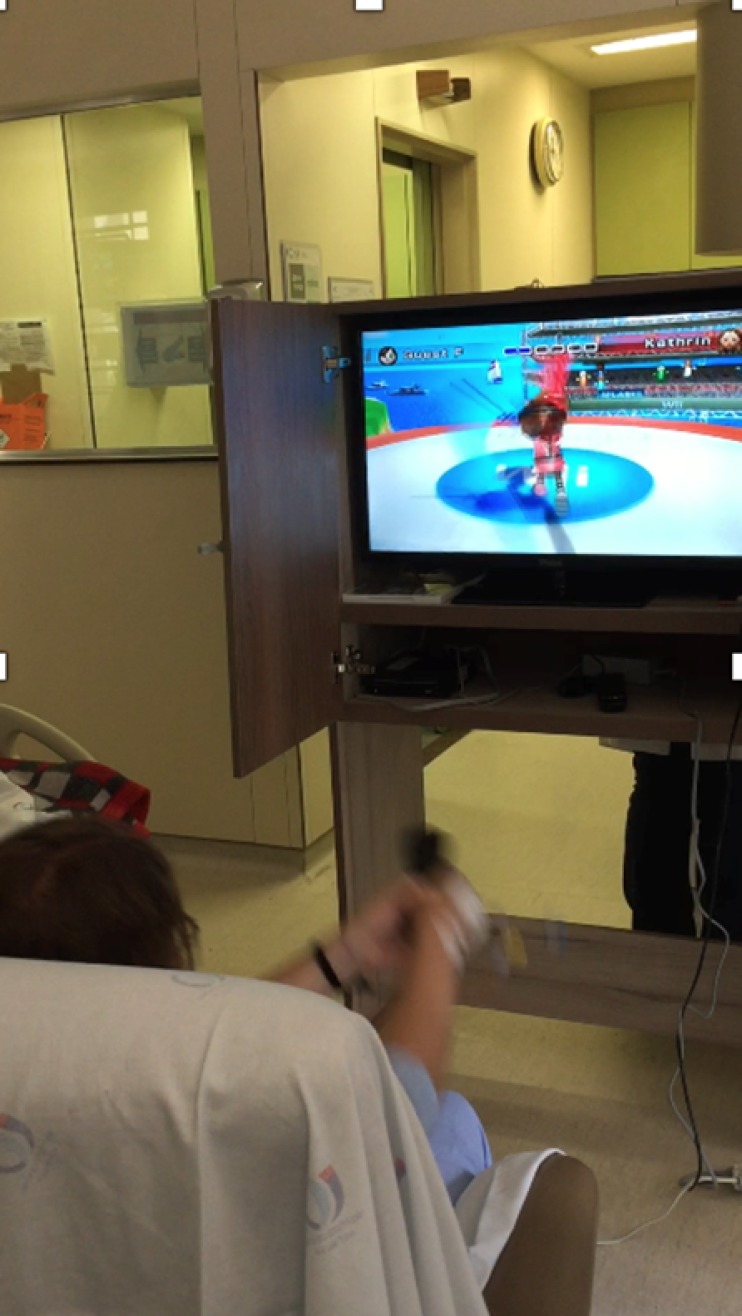
Patient sitting on a chair during the physiotherapy session playing a swordfighting game.

The Nintendo Wii^TM^ (Nintendo of America Inc.^TM^, USA) gaming system was used for the sessions, and the game was shown on a television screen (Figure 1). The games used in the study were divided into two categories: (1) patients who were unable to stand played a swordfighting game and a table tennis game from a sitting position in a bed or a chair (Figure 2), and (2) patients who could get out of the ICU bed and remain in a standing position played a game that required the patient to move his or her legs and deflect or jump over obstacles and a game that required the patient to balance on a ball and juggle with balls. Each patient played the games for six minutes.

The patients signed an informed consent form, and then we placed two accelerometers one on the wrist and the other on ankle of the included patients prior to the videogame sessions. The game's start and finish times were noted. Immediately before the game began, the vital signs, use of catheters, probes or other equipment, and level of the patient's fatigue were recorded. The intervention was then performed with the patient, and if necessary, the intervention could be stopped. During the intervention, the level of activity was evaluated by the use of the accelerometer and the Borg scale. Immediately after the game ended, all variables previously mentioned were re-evaluated, and the patient was asked to complete a "satisfaction with activity" questionnaire.

#### Statistical analysis

The analysis was performed using SigmaStat (version 3.0). The Kolmogorov-Smirnov test was used to verify the normality of the data, which were presented as the mean ± standard deviation or the median and interquartile range. The absolute number and percentage were also used. Categorical variables were presented as the absolute (relative) number.

For analysis of the activity level, the duration of time spent at each level of physical activity during the videogame session was calculated. Patient satisfaction regarding the videogame sessions was presented qualitatively.

## RESULTS

During the study period, 100 physical therapy sessions were analyzed. Over nine months, 68 patients met the inclusion criteria for the study. However, seven patients were excluded because they could not follow verbal commands and were not able to play the games. Therefore, 60 patients received the intervention. Since the patients were allowed to participate in the videogame sessions more than once, we evaluated the data of each session.

The mean age of the patients was 47 ± 17 years old, 50% of the participants were male, and the mean SAPS III score was 48 (14.5). In nine sessions, patients were under IMV during the videogame session; in eight sessions, patients used an orotracheal tube; in three sessions, patients had a tracheotomy; and in 16% of the sessions, patients were using vasoactive drugs. These data are described in table 1. The number of sessions in which patients were using an invasive device is also shown in table 1.

**Table 1:** Population characteristics (n = 60 patients and 100 sessions)

Variables	
Age, years old	47 ± 17
SAPS III	53 ± 14
Male sex	30 (50)
Cause of patients' ICU admission	
Respiratory	12 (20)
Nonrespiratory	48 (80)
Use of invasive devices during session	
Nasal oxygen cannula	37 (37)
Feeding tube	72 (72)
Central venous catheter	37 (37)
Peripheral venous catheter	59 (59)
Drain	7 (7)
Dialysis catheter	4 (4)
Use of vasoactive drugs during session	16 (16)
Use of tracheostomy during session	3 (3)
Use of orotracheal tube during session	8 (8)
Use of IMV during session	9 (9)

SAPS III - Simplified Acute Physiology Score III; ICU - intensive care unit; IMV - invasive mechanical ventilation. Results expressed as the mean ± standard deviation or n (%).

### Assessment of the level of activity

The videogame elicited a light level activity for all patients and a moderate level of activity for all but one patient. Table 2 describes the mean duration at each level of activity in physical therapy. Of the 100 sessions, 14 were performed standing. During the videogame therapy, a light level of activity was reached 59% of the time, and a moderate level of activity reached 38% of the time. A vigorous level of activity was reached in 12 of the 100 sessions, and a very vigorous level of activity was reached in six sessions; of these six sessions, four sessions were performed while the patient was standing on a platform (Table 2). The modified Borg scale score before the videogame session was 0; after the videogame session, the median modified Borg scale score was 2 (0 - 4), which indicates light to moderate respiratory fatigue (Table 2).

**Table 2:** Level of activity characteristics (n = 100 sessions)

Modified Borg scale	
Initial score	0 (0 - 0)
Final score	2 (0 - 4)
Percentage of time spent in each level of activity	
Light (n =100 sessions)	59 ± 21
Moderate (n = 99 sessions)	38 ± 21
Vigorous (n = 12 sessions)	16 ± 9
Sessions performed in standing position (n = 12)	4 (4)
Sessions performed in sitting position (n = 12)	8 (8)
Very vigorous (n = 6 sessions)	8 ± 6

Results expressed as the median (25% -75% quartiles), mean ± standard deviation or n (%).

### Safety

No adverse events or accidental removals of the invasive devices occurred during the videogame sessions, and there were no significant changes in vital signs during the sessions. Only 2% of participants reported discomfort during the sessions, which was described as dizziness.

### Satisfaction

Regarding the patients' acceptance and satisfaction with the videogame sessions, the patients reported that they enjoyed the activity and that it was an activity they could accomplish given their physical status. On a scale from 0 to 10, the patients gave a median score of 10 for how much they liked the videogame session. A total of 86% of the patients reported that they would like to play the videogame in their future physical therapy sessions. These results are shown in table 3.

**Table 3:** Evaluation of the activity (n = 100 sessions and 60 patients)

Do you think you are able to play this game?	
Yes	86 (86)
No	1 (1)
Did not answer	13 (13)
On a 0 to 10 scale, how much did you enjoy this activity?	10 (8 - 10)
Do you prefer the videogame session or other activities during physical therapy?	
Videogame	22 (32)
Videogame and deambulation training	7 (7)
Standard exercises and deambulation training	4 (4)
Walking	9 (9)
All exercises	24 (24)
Did not answer	23 (23)
Would you like to take on this activity in the next physical therapy session?	
Yes	84 (84)
No	2 (2)
Did not answer	14 (14)
Did you feel any discomfort during this activity?	
Dizziness	2 (2)

Results expressed as n (%) or median (25% - 75%).

## DISCUSSION

This study aimed to evaluate the safety and practicality of virtual reality as a rehabilitation tool for patients in the ICU. Additionally, we assessed the level of physical activity elicited by patients using this tool. No adverse events with potential risks occurred during the exercise sessions in which the video game was used, and no device was inadvertently removed. Our results also showed that physical therapy using the videogame was able to elicit light and moderate levels of activity in patients and in some patients, a vigorous level of activity. A brief report of a few cases in the literature described the use of virtual reality in the ICU.^(20)^ In a subsequent study, a series of cases showed that this tool can be feasibly implemented in the ICU.^(26)^ Our study contributes to the literature by providing data from a large population of patients in the ICU, including feasibility and safety data, as well as accelerometry data, showing the levels of activity that were safely elicited by the patients.

The safety criteria recommended for exercises in the ICU are well established in the literature.^(28)^ In a study that evaluated the safety of activities in bed versus those out of bed, it was noted that the frequency of potential events was not different. The potential events that occurred more frequently were physiological changes that were usually transient and were resolved after rest without any interventions.^(24)^ A study evaluating the safety of mobilization and ambulation in the ICU reported the occurrence of potential safety events in only 1% of patients.^(3)^ Other mobility programs and some specific therapies, such as cycloergometers, have been shown to be safe and practical.^(14-16)^ We did not observe any intercurrence in the video game exercise with the equipment or changes in vital signs that required interruption of the session or caused an injury to the patient. We had no intercurrence of access, drains, or probe loss, even in patients on mechanical ventilation, which suggests that virtual reality is safe for ICU patients. Additionally, several studies have suggested the need for new exercise tools for increased mobilization and physical activity in ICU patients with the aim of reducing sedentary behavior during hospitalization.^(11-16)^ Our results showed that body movements performed in virtual reality were able to generate sufficient levels of activity, offering some degree of exercise and changing the patients' state of immobility, and these results address the demands in the literature for new tools that safely reduce immobilization in ICU patients.^(35,36)^

These findings are important because for patients in the ICU, physical activity has shown benefits in preventing immobilization syndrome, which can have a negative impact on patients' daily activities after discharge from the ICU.^(1-3)^ In other populations, this tool has shown promise for rehabilitation and the continuation of exercises in patients with a variety of diseases, and it has been shown that games can produce a sufficient level of body movement to encourage healthy individuals to participate in an activity and in rehabilitative processes.^(18,19,37-39)^ According to the American College of Sports Medicine, the intensity of activity is one factor that needs to be established for an exercise prescription.^(40)^ In this study, we found that the Nintendo Wii^TM^ could safely elicit light- to moderate-intensity exercise in ICU patients. We also found that physical therapy with videogames was well accepted by the patients, as most patients enjoyed it and would like to participate in the activity in the next session. More studies have been conducted using virtual reality because of the motivation that this tool can provide for patients. In a study that evaluated the benefits and challenges of this new tool in physical therapy, the authors found that the patients have fun with this tool^(25,38,40)^ and develop the motivation to perform an activity.^(21,23,25)^

A limitation of the present study was that it was a unicentric study and was carried out in a single ICU. We did not separate surgical and clinical patients. Additionally, we performed the intervention with a few mechanically ventilated patients. Thus, more studies with patients using IMV, more studies that explore other variations of exercises in this specific population, such as different frequencies of the intervention, and more clinical trials that use controlled groups for a better description of the benefits of this type of intervention are needed. To our knowledge, our study is the first to analyze the level of activity elicited by ICU patients. With our data, we can show the objective value of specific equipment by identifying and classifying the types of activity elicited by individuals, such as those described in the literature, for the analysis of physical activity.

## CONCLUSION

The Nintendo Wii^TM^ gaming system elicited light to moderate levels of activity in intensive care unit patients, with the possibility of reaching a vigorous level of activity. It was also observed that the Nintendo Wii^TM^ gaming system is a safe tool and is likely to be chosen by patients during physical therapy and rehabilitation in this environment.
